# Establishing the Approach to the Diagnosis of Hemolytic Anemia in the Genetic Era: A Case Series

**DOI:** 10.7759/cureus.67952

**Published:** 2024-08-27

**Authors:** Aayushi Guru, Pratibha Meena, G K Sawke, Sakshi Tripathi

**Affiliations:** 1 Pathology, Atal Bihari Vajpayee Government Medical College Vidisha, Vidisha, IND

**Keywords:** diagnosis, blood, approach, anemia, hemolytic

## Abstract

Background: Hemolytic anemia is characterized by the premature destruction of red blood cells, a condition that ranges from chronic to life-threatening. Hereditary hemolytic anemias (HHAs) encompass a broad spectrum of disorders including hemoglobinopathies, enzymopathies, and membrane disorders. In India, hemoglobinopathies, notably thalassemia and sickle cell disease, are significant health concerns contributing to high morbidity and mortality rates. Despite many cases being clinically insignificant, these disorders exert a considerable public health burden due to their prevalence. Techniques like next-generation sequencing (NGS) and high-performance liquid chromatography (HPLC) have emerged as powerful tools for identifying and diagnosing HHAs. NGS enables comprehensive genetic analysis, pinpointing mutations associated with hemoglobinopathies and other forms of hereditary anemia. HPLC allows precise quantification and characterization of hemoglobin variants, which is crucial for diagnosing hemoglobinopathies.

Aims and objectives: This study aimed to establish a refined approach for diagnosing hemolytic anemias and categorize different types of hemolytic anemia using state-of-the-art technologies for early and precise treatment interventions.

Materials and methods: This retrospective study was conducted in the Hematology Section of the Department of Pathology at Atal Bihari Vajpayee Government Medical College, Vidisha, Madhya Pradesh. The study included six patients diagnosed with hemolytic anemia based on comprehensive hematological, biochemical, and molecular evaluations.

Results: The retrospective analysis of six cases of hemolytic anemia highlighted the diagnostic approach utilized. Clinical presentations, physical examinations, routine hematological investigations, advanced diagnostic modalities, and hemoglobin electrophoresis were instrumental in identifying specific types of hemolytic anemias.

Conclusion: Despite the availability of advanced diagnostic techniques, basic hematological investigations remain the cornerstone in the initial evaluation of HHAs. Hemoglobin electrophoresis plays a pivotal role in confirming diagnoses. In some cases, subtle hematological findings necessitate thorough evaluation, including familial studies, to guide appropriate management strategies.

## Introduction

Hereditary hemolytic anemias (HHAs) are a group of disorders characterized by intrinsic defects in red blood cells, leading to increased destruction of erythrocytes, hyperbilirubinemia, and erythroid hyperplasia. This group includes conditions such as defects in the red cell membrane (e.g., hereditary spherocytosis, elliptocytosis), abnormal hemoglobin synthesis (e.g., sickle cell anemia), impaired globin synthesis (e.g., thalassemia), and deficiencies in erythrocyte enzymes (e.g., G6PD deficiency, pyruvate kinase deficiency). These disorders represent a significant cause of mortality and morbidity in developing countries, ranking second only to infection and malnutrition. Diagnosing HHAs can be challenging due to their varied presentations. It is crucial to first identify patients with suspected HHAs and gather data on hemolysis. Hemolysis in HHAs is characterized by increased hemoglobin breakdown, leading to unconjugated hyperbilirubinemia manifested clinically by jaundice, elevated lactate dehydrogenase (indicative of cellular destruction), and reticulocytosis, which is the bone marrow's compensatory response to red blood cell loss [1-5]. Hematological, biochemical, and laboratory findings play a pivotal role in determining the appropriate diagnostic workup for accurate diagnosis and timely treatment. Despite their relative rarity, hemolytic anemias pose significant diagnostic hurdles for hematologists and clinicians. This summary aims to provide valuable insights and clarify the diagnostic approach for HHAs, facilitating tailored treatment strategies based on the underlying cause.

## Materials and methods

This institution-based study aims to establish an effective diagnostic approach for hemolytic anemia and categorize these conditions using advanced technologies to ensure early and optimal treatment outcomes.

The present study is conducted in the Hematology Section of the Department of Pathology, A.B.V.G.M.C Vidisha, Madhya Pradesh. All cases of hemolytic anemia that were diagnosed in the department from October 2023 to April 2024 were included in the present retrospective study. This retrospective study includes all cases of hemolytic anemia diagnosed from October 2023 to April 2024. All newly diagnosed cases of hemolytic anemia were included in the study. Already known cases of hemolytic anemia were excluded from the study. Six patients with hemolytic anemia underwent comprehensive hematological, biochemical, and molecular assessments as part of routine blood investigations.

A detailed history of the patient, including name, age, gender, presenting complaints, past history, family history, and transfusion history, was taken. EDTA anticoagulated blood samples were analyzed in a fully automated counter in the hematology Laboratory of the Department of Pathology. A multiparameter hemogram comprising hemoglobin, hematocrit, MCV, MCH, MCHC, RDW, RBC count, WBC count, platelet count, and reticulocyte count using supravital stain was done. Leishman stained peripheral blood smears were analyzed. Differential leukocyte count and red cell morphological alteration (hypochromasia, anisopoikilocytosis, sickle cells, target cells, spherocytes, polychromasia, nucleated red cells) were noted. Based on clinical history, examination findings, and preliminary laboratory results, further investigations were conducted to diagnose hemolytic anemias. The serum samples were also analyzed for biochemical parameters like serum bilirubin, LDH, and uric acid. Special diagnostic tests like the sickling test by using sodium metabisulfite solution as a reducing substance and the Coombs antiglobulin test using red cell suspension were done when needed. Type and quantification of various hemoglobin variants were performed by fully automated cation exchange high-performance liquid chromatography (HPLC) using BIORAD VARIANT II (Bio-Rad Laboratories, Inc., Hercules, USA). Results of HPLC were correlated with the clinical profile and family studies.

This summary seeks to contribute valuable information on a topic underrepresented in medical literature, aiming to elucidate the diagnostic approach and guide tailored treatment strategies based on specific causative.

## Results

Case 1

A 43-year-old male presented to the medicine department with complaints of weakness, lethargy, and difficulty in breathing. His blood sample was sent to hematology for CBC and PS examination, which showed hemoglobin of 5.8g/dl (reference range: 14-17 mg/dl), hematocrit 19% (reference range: 42-52%), MCV 82.10 fl (reference range: 75-98 fl), MCH 26.2 pg (reference range: 26-33 pg), MCHC 30.4 g/dl (reference range: 33-37 g/dl), RDW-CV 25% (reference range: 11-15%), and TLC 14.2x109/L with peripheral smear showing marked degree of anisopoikilocytosis with severe hypochromasia. Red cells are predominantly macrocytic, good number of schistocytes, few tear drop cells, and polychromatophilic cells are seen as suggestive of a hemolytic blood picture. Biochemical investigations revealed LDH 350U/L and total serum bilirubin 2.2 mg/dl. Peripheral smear showed significant schistocytes which was the diagnostic dilemma, and further workup for trauma, infectious diseases, and microangiopathic hemolytic anemia (MAHA) was made to rule out possible differential diagnosis. Upon tracing history, the patient underwent cardiac prosthesis and valve replacement surgery ten months back, with no symptoms of infections and normal clotting assays. Echocardiography for valve replacement further suggested a malfunction and a diagnosis of mechanical Hemolytic anemia was made.

Case 2

**Figure 1:**
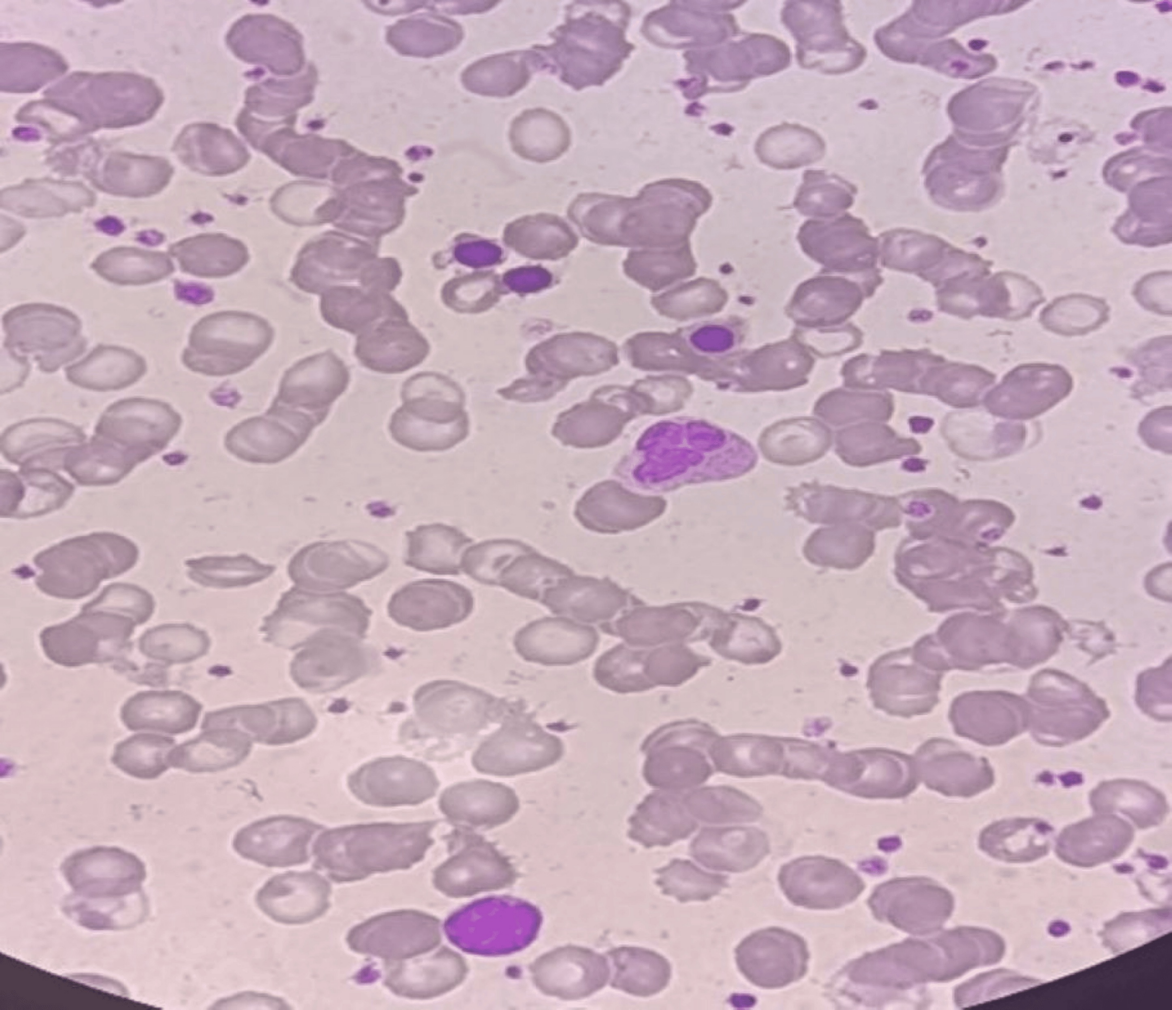
Peripheral smear of a seven-year male child showing nucleated RBCs

A seven-year-old male child was admitted to the PICU with complaints of weakness, reluctance to play, and delayed milestones requiring multiple blood transfusions. A blood sample was sent to hematology for routine CBC and PS examination. His CBC showed hemoglobin of 5.2 g/dl (reference range: 14-17 mg/dl), MCV 102.1 fl (reference range: 75-98 fl), MCH 24.4 pg (reference range: 26-33 pg), MCHC 36.2 g/dl (reference range: 33-37 g/dl), RDW-CV 20% (reference range: 11-15%), and TLC 75x109/L with distribution as neutrophils 80%, lymphocyte 15%, monocytes 4%, eosinophils 1% and nil basophils. Peripheral smear revealed predominantly microcytic hypochromic red cells along with target cells, tear drop cells, polychromatophilic cells, and nucleated red blood cells (190/100 WBC) with a mild left shift of myeloid series suggestive of leucoerythroblastic blood picture (Figure 1) and the possibility of hemolytic anemia. In view of microcytic hypochromic and target cells in a pediatric child, HPLC was performed along with a workup of parents, on which a diagnosis of beta-thalassemia major was reported. 

Case 3

**Figure 2:**
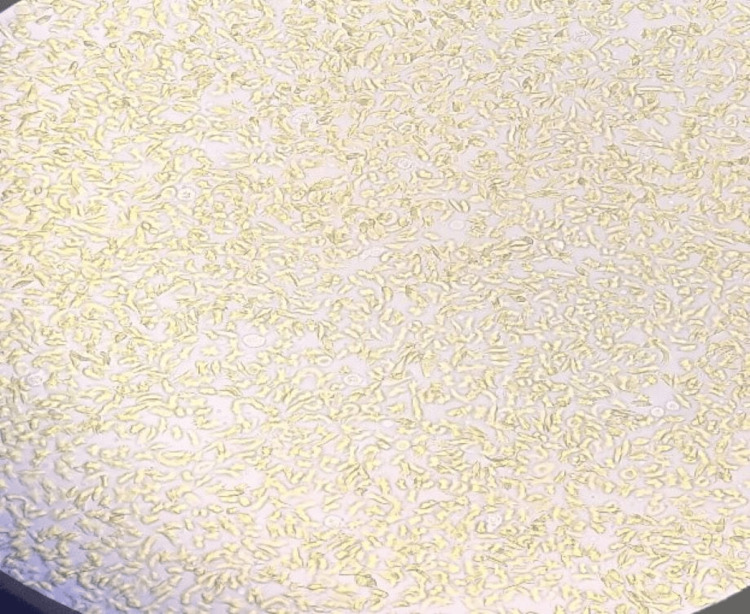
Sickling test- positive

A 25-year-old male was admitted to the Department of Medicine with body pain and weakness. A blood sample was sent to hematology for routine CBC and PS examination. His CBC showed hemoglobin of 4g/dl (reference range: 14-17 mg/dl), MCV 90.2fl (reference range: 75-98 fl), MCH 26.0pg (reference range: 26-33 pg), MCHC 28.4 g/dl (reference range: 33-37 g/dl), RDW-CV 20% (reference range: 11-15%), TLC 18x109/L, and platelet count of 180x109 /L (reference range: 150-400x10 9/L). Biochemical investigation revealed serum bilirubin 1.3mg/dl and LDH 225U/L. Peripheral smear showed moderate anisopoikilocytosis with macrocytic red cells, target cells, sickle cells and polychromatic cells raising the possibility of hemolytic anemia (likely sickle cell anemia). A sickling test was performed to rule out sickle cells, which was positive and the diagnosis of sickle cell anemia was confirmed (Figure 2).

Case 4

An 11-month-old male child was admitted to the NICU with weakness, diarrhea, and requiring multiple blood transfusions since birth. On examination, the medicine resident reported hepatomegaly. A blood sample was sent to the hematology department for routine CBC and PS examination. His CBC showed hemoglobin 8.2 g/dl (reference range: 14-17 mg/dl), MCV 80.4 fl (reference range: 75-98 fl), MCH 26.2pg (reference range: 26-33 pg), MCHC 34.4g/dl (reference range: 33-37 g/dl), RDW-CV 20% (reference range: 11-15%), platelet count 80x109/L (reference range: 150-400x10 9/L) and TLC within the normal range. Peripheral smear reveals the marked degree of anisopoikilocytosis with dimorphic red cells and marked hypochromasia, schistocytes (> 1%), polychromatic cells, and few nucleated RBCs. A significant number of schistocytes with thrombocytopenia needed a workup for trauma and thrombotic microangiopathy (TMA) and other non-TMA causes. The triad of schistocytes (>1%), anemia, and thrombocytopenia with a preceding history of diarrhea made the possibility of Microangiopathic hemolytic anemia followed by infections, which was further confirmed by bacterial culture in Microbiology. 

Case 5

**Figure 3:**
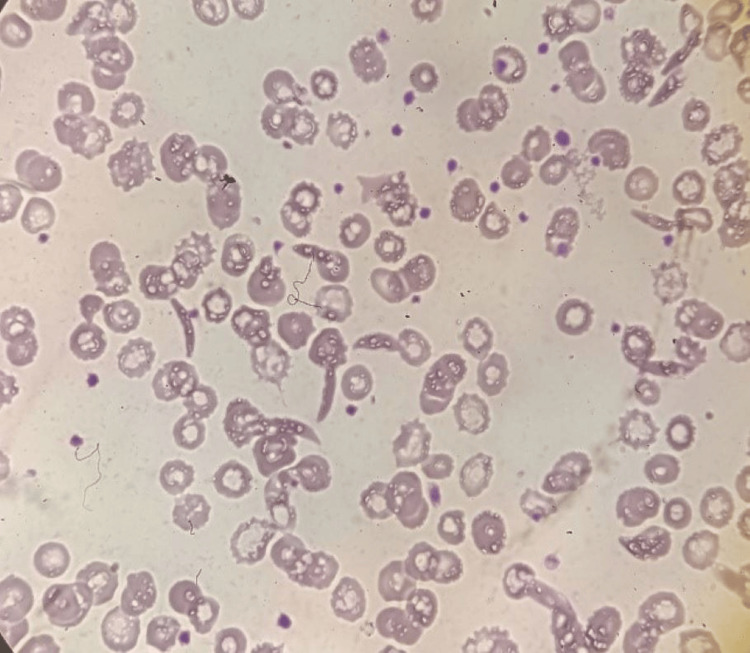
Peripheral smear showing marked anisopoikilocytosis with sickle cells

A 29-year-old pregnant female was admitted to the obstetrics department with G2P1L0A1 at 31 weeks gravida with difficulty in breathing. A blood sample was sent to the hematology department for routine CBC and PS examination. CBC revealed hemoglobin 6.2 mg/dl (reference range: 14-17 mg/dl), MCV 92.4 fl (reference range: 75-98 fl), MCH 24.0 pg (reference range: 26-33 pg), MCHC 32.0g/dl (reference range: 33-37 g/dl), RDW CV 22.5% (reference range: 11-15%), TLC 21x109/L, and platelet count 300x109/L (reference range: 150-400x10 9/L). A peripheral smear was performed, which showed a marked degree of anisopoikilocytosis, with macrocytic red cells and marked hypochromasia. Red cells also showed a significant number of sickle cells, schistocytes, and polychromatic cells. Biochemical investigation revealed LDH 180U/L. To rule out the presence of sickle cells, a sickling test and HPLC were performed, which was observed as positive HbS (Figure 3).

Case 6

A seven-year-old male child was brought to the outpatient department with complaints of lethargy, dullness, and loss of interest in play and studies. His blood sample was sent for routine CBC which showed hemoglobin 8.6 mg/dl (reference range: 14-17 mg/dl), MCV 64 fl (reference range: 75-98 fl), MCH 22.4 pg (reference range: 26-33 pg), MCHC 34.2 g/dl (reference range: 33-37 g/dl), RDW-CV 16% (reference range: 11-15%) and platelet count 90x109/L (reference range: 150-400x10 9/L). In view of low hemoglobin and platelet count, a peripheral smear was examined and showed a microcytic hypochromic blood picture with numerous target cells, tear drop cells, and a manual platelet count of 100x109/L. Microcytic hypochromic anemia with target cells suggested the possibility of thalassemia which was confirmed with HPLC and the child was reported as beta-thalassemia minor.

## Discussion

When a patient presents with anemia, a stepwise approach should be followed. Initial simple investigations will first alert the physician to the suggestion of hemolysis as the cause of the anemia. These include a normo /macrocytic/microcytic anemia, raised reticulocyte count, raised unconjugated bilirubin, reduced haptoglobin, and blood smear with polychromasia or more specific features, such as spherocytes, schistocytes or agglutination. Autoimmune hemolytic anemia (AIHA) patients may develop reticulocytopenia as a result of parvovirus B19 infection or bone marrow invasion by a lymphoproliferative illness. Membrane deficiencies or repetitive minor membrane losses by macrophages are the causes of spherocytes.

Spherocytosis is not diagnostic for hemolytic anemia because both hereditary spherocytosis and immune etiologies (e.g., AIHA, drug-induced immune hemolytic anemia) may cause spherocytes. Fragmented cells known as schistocytes are the end product of intravascular destruction that takes place in MAHA disorders. Bite and blister cells arise due to incomplete phagocytosis and are associated with oxidative factors including a deficit of glucose-6-phosphate dehydrogenase (G6PD) [6-8]. The direct antiglobulin test helps distinguish immunological and non-immune causes of hemolytic anemia. Urinary hemosiderin, red cell fragments on smear, or a markedly elevated LDH all point to a mostly intravascular hemolytic process. Once hemolysis is confirmed, further investigation is needed to establish whether that hemolysis is hereditary or immune.

Accurately diagnosing HHAs is challenging because their clinical features can overlap with those of other conditions, even when the underlying causes are distinct. Therefore, genetic testing is recommended in the following scenarios: (i) when routine studies fail to identify the causative mechanism or yield inconclusive results; (ii) when recent transfusions have introduced mixed RBC populations that can interfere with biochemical and other tests; and (iii) when diagnosing a newborn [9]. With advancements in understanding the genetic mechanisms regulating RBC function, genetic testing is increasingly utilized to confirm HHA diagnoses worldwide. Historically, molecular diagnosis relied on Sanger sequencing of individual genes [10-12]. However, targeted gene panels are now commonly used for routine molecular diagnosis, offering advantages over whole exome or genome sequencing. These include lower cost, faster turnaround time, simpler data analysis, better coverage of relevant regions, and fewer incidental findings [13].

Inherited hemoglobinopathies, such as sickle cell disease and thalassemia, may present at birth or later, depending on their severity. These conditions should always be considered when evaluating a child with hemolysis, as detailed in previous reviews in the American Family Physician [14-15].

**Figure 4:**
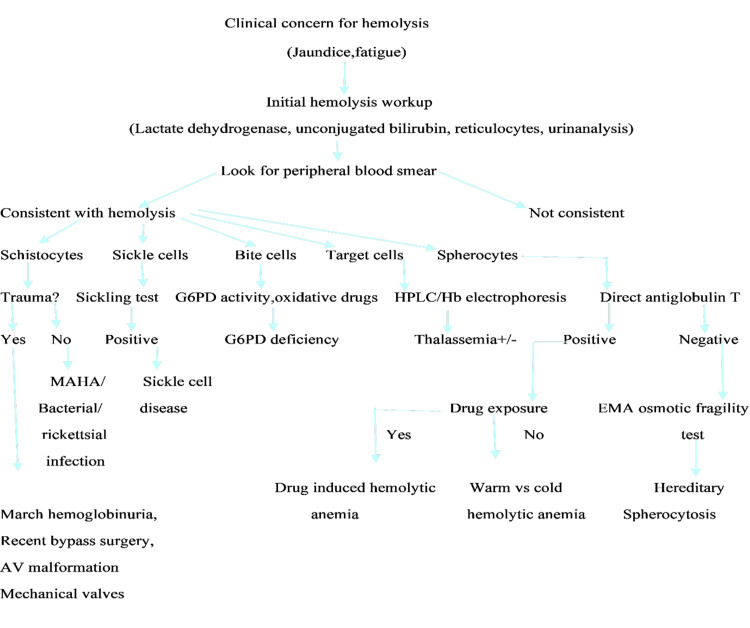
Approach to the diagnosis of hemolytic anemia G6PD deficiency: Glucose 6 phosphate deficiency; HPLC: high-performance liquid chromatography; MAHA: microangiopathic hemolytic anemia; AV: arteriovenous; EMA: eosin-5-maleimide acid

 The following approach has been used in the further investigation of patients to establish the cause of hemolysis (Figure 4). 

## Conclusions

The numerous confounding variables in the clinical setting pose difficulty in diagnosing hemolytic anemia, which causes confusion among physicians. On the other hand, the pathophysiologies of many HHAs are now well understood due to technological advancements and updated diagnostic procedures. Still, the gold standard for hemolytic anemia diagnosis is a thorough history and physical examination. In summary, the adoption of molecular technologies such as NGS along with HPLC as primary diagnostic tools marks a significant advancement in hematology. This is particularly vital in regions like India, where hereditary hemolytic anemias pose substantial health challenges, enabling more effective identification and management of these complex disorders. Even though developing nations have recently implemented a number of positive initiatives, screening tests and premarital counseling for the management of inherited hemolytic anemias are typically not given the priority they need.
